# The Biographical Dictionary of the Society for the Diffusion of Useful Knowledge

**Published:** 1842-10

**Authors:** 


ART. XI.—*The Biographical Dictionary of the Society for Diffusing Useful Knowledge.* Vol. I. Part I. A.. —*London*, 1842. 8vo, pp. 440.

WE were induced to look into this new Dictionary by the hope of finding the *medical lives* more carefully written than we had found to be the case in other works of a somewhat similar kind; and we were not disappointed. All the lives of physicians and surgeons which we have examined are composed in a simple and unaffected style, and contain, in short compass, all the more material facts connected with the individual, and a correct list of his writings. The lives of the ancient physicians display great learning and research on the part of the author; and we doubt not but that most of our readers will be introduced, in his pages, to the acquaintance of not a few professional brethren heretofore unknown to them. The modern lives are compiled with equal care, and comprehend the names of almost every man, in every country, who has been in any way distinguished in medical science or practice. We strongly recommend this work to the members of our profession as one which, when complete, will contain a fuller and truer history of the lives and writings of medical men than any other that has yet appeared; and we believe we may conscientiously recommend it to the members of all other professions on the same grounds of superiority.

